# Potential of Farnesyl Transferase Inhibitors in Combination Regimens in Squamous Cell Carcinomas

**DOI:** 10.3390/cancers13215310

**Published:** 2021-10-22

**Authors:** Linda Kessler, Shivani Malik, Mollie Leoni, Francis Burrows

**Affiliations:** Kura Oncology, Inc., San Diego, CA 92130, USA; linda@kuraoncology.com (L.K.); smalik@kuraoncology.com (S.M.); mleoni@kuraoncology.com (M.L.)

**Keywords:** HNSCC, farnesyl transferase, tipifarnib, combination regimen

## Abstract

**Simple Summary:**

Current therapies for recurrent and metastatic squamous cell carcinomas (SCCs) are associated with poor patient outcomes, and options for later lines of treatment are very limited. In cases where single-agent therapy may be insufficient to eradicate the tumor, thus allowing outgrowth of resistant cells, a well-chosen combination of therapeutic agents may enable improved outcomes. Tipifarnib, a farnesyl transferase inhibitor, is a small molecule drug candidate that has demonstrated promising clinical activity in HRAS-mutant head and neck squamous cell carcinoma (HNSCC). New molecular analyses suggest that HRAS may also be important in some HNSCC cases where it is not mutated, which might allow tipifarnib to be active in a broader population of HNSCC patients when used in combination with other agents such as cisplatin, cetuximab, or alpelisib. Other non-HRAS oncoproteins that can also be blocked by tipifarnib may offer alternative approaches to combination regimens for SCCs.

**Abstract:**

Current therapies for recurrent and metastatic SCC are associated with poor outcomes, and options for later lines of treatment are limited. Insights into potential therapeutic targets, as well as mechanisms of resistance to available therapies, have begun to be elucidated, creating the basis for exploration of combination approaches to drive better patient outcomes. Tipifarnib, a farnesyl transferase inhibitor (FTI), is a small molecule drug that has demonstrated encouraging clinical activity in a genetically-defined subset of head and neck squamous cell carcinoma (HNSCC)–specifically, tumors that express a mutation in the *HRAS* protooncogene. More recently, bioinformatic analyses and results from patient-derived xenograft modeling indicate that HRAS pathway dependency may extend to a broader subpopulation of SCCs beyond HRAS mutants in the context of combination with agents such as cisplatin, cetuximab, or alpelisib. In addition, tipifarnib can also inactivate additional farnesylated proteins implicated in resistance to approved therapies, including immunotherapies, through a variety of distinct mechanisms, suggesting that tipifarnib could serve as an anchor for combination regimens in SCCs and other tumor types.

## 1. Tipifarnib in HRAS-Mutant HNSCC—History, Preclinical Validation, and Clinical Development 

The RAS family is a group of low molecular weight guanosine triphosphate (GTP)-binding proteins localized to the cell membrane that play a pivotal role in the transduction of cell growth-stimulating signals. Well-established effectors of RAS are the protein kinase RAF and the lipid kinase PI3-kinase (PI3K). Following recruitment by RAS to the plasma membrane and activation by phosphorylation, RAF induces a phosphorylation cascade that drives the transcription of genes associated with cell proliferation [1]. PI3K activation leads to increased cell motility, invasiveness, and suppression of apoptosis [2,3]. RAS-driven downstream effector pathways also regulate the cell cycle and integrin signaling [4,5].

Approximately 30% of human tumors express a mutation in one of three *RAS* protooncogenes (*KRAS*, *NRAS*, and *HRAS*) encoding four RAS proteins (KRAS4A, KRAS4B, NRAS, and HRAS) [6]. The frequency of *RAS* mutation and the dominant isoform vary depending on the tissue and tumor type [7]. The majority of these mutations are localized to codons 12, 13, or 61 and defined as “activating mutations” because they encode RAS proteins with suppressed GTPase activity that allows RAS to remain in the GTP-bound active state [8,9]. The critical role of RAS in oncogenic transformation was characterized by expression of dominant-negative forms of RAS and homologous recombination to disrupt mutated, active *RAS* genes in various human cancer cell lines [10,11].

RAS isoforms must associate with the inner surface of the plasma membrane to transduce extracellular signals. To become active, RAS undergoes several post-translational modifications. The first step is the farnesylation of the cysteine in the CAAX box at the C-terminal end (where C represents cysteine, A represents an aliphatic amino acid, and X represents any amino acid) [6]. The enzyme farnesyltransferase (FTase) recognizes the CAAX motif and transfers a 15-carbon farnesyl isoprenoid from farnesyl diphosphate to the cysteine residue. The AAX amino acids subsequently are cleaved by RAS-converting enzyme I, and the farnesylated cysteine is carboxymethylated by isoprenylcysteine carboxyl methyltransferase [12]. Further palmitoylation (KRAS4A, NRAS, and HRAS or the presence of a polybasic domain (KRAS4B) leads to anchoring of the protein in the plasma membrane [13]. 

With the elucidation of this RAS post-translational modification pathway in the late 1980s, FTase became a viable pharmacological target to affect RAS function in cancer. Preliminary strategies were directed towards CAAX tetrapeptide inhibitors, which were competitive with the protein substrate [14]. However, such tetrapeptides were not efficiently taken up into cells, and the drug discovery efforts shifted toward more stable, peptidomimetic inhibitors [15,16,17,18]. Small molecule inhibitors were identified through high-throughput screening efforts and aided by crystallographic structures [19]. One such drug candidate, which later advanced into clinical evaluation, was R115777, also known as tipifarnib, a heterocyclic non-peptidomimetic that inhibits the FTase prenylation of KRAS in vitro with an IC_50_ of 7.9 nM [20]. 

Tipifarnib was the first FTI to enter clinical development in 1997, and its safety and efficacy have been assessed in more than 70 clinical studies [21,22,23,24,25]. Observations that mutations within *KRAS* are most common in lung, colorectal, and pancreatic tumors; *NRAS* mutations typically observed in human myeloid cancers; and *HRAS* mutations found in bladder, thyroid, and head and neck tumors [8] helped guide the clinical development program. However, Phase 3 trials in non-enriched patient populations resulted in no significant antitumor effect in patients with advanced colorectal cancer [26]. In addition, no significant increase in response rate was observed in patients with pancreatic carcinoma when tipifarnib was combined with gemcitabine [27]. Overall, tipifarnib failed to achieve clinically meaningful improvements in two solid tumors known to highly express mutations in *KRAS*. Subsequently, it was discovered that certain farnesylated proteins—including KRAS and NRAS—can be rescued from membrane displacement in the presence of FTIs by an alternative prenylation by the enzyme geranylgeranyltransferase (GGTase) [28,29]. Conversely, the third family member, HRAS, is not a GGTase substrate, and therefore its membrane localization and cellular function are diminished by FTIs [29]. Thus, it was hypothesized that using tipifarnib to target enriched patient populations of tumors harboring *HRAS* mutations via a classical precision medicine approach might yield more favorable clinical outcomes. 

Despite being comparatively less frequent than those of *KRAS* and *NRAS*, mutations in *HRAS* are highly expressed in follicular thyroid cell-derived and in medullary thyroid carcinomas, as well as in head and neck and bladder cancers [30,31,32,33,34]. In a dedifferentiated thyroid cancer model, Untch et al. demonstrated that mice harboring flox-and-replace HRAS^G12V^ and floxed p53 alleles developed aggressive tumors and 50% mortality after 40 weeks [35]. Treatment of these mice with tipifarnib significantly improved survival and reduced tumor volume relative to vehicle-treated controls at 14 days. However, a subset of mice presented persistent, albeit diminished, tumor growth, occurring despite appropriate HRAS defarnesylation, suggesting an adaptive response to FTI treatment. To confirm this hypothesis, the investigators treated human and murine *HRAS* mutant cell lines with tipifarnib and observed increased GTP loading of wild-type KRAS and NRAS, a mechanism by which the efficacy of blunting oncogenic HRAS signaling could be circumvented. The authors further demonstrated that prolonged treatment of *HRAS* mutant tumors with tipifarnib elicited the emergence of nonsense mutations in *Nf1,* which encodes a GTPase-activating protein that is a negative regulator of RAS. Notably, loss of function mutations in *Nf1* have also been shown to confer resistance to other therapies for melanoma and lung cancer [36,37,38]. In addition to the *Nf1* loss, an activating mutation was found in *Gnas*, a complex locus whose most well-characterized transcript is the stimulatory G-protein alpha subunit (G_αs_). Similar findings have been described in the resistance to RAF inhibitors in melanoma cells, indicating that this mechanism may be common in cells that are dependent on cAMP for differentiated function [39]. Collectively, these adaptations to HRAS inhibition may limit the effectiveness of therapies for certain patients with *HRAS* mutant malignancies, although other concurrent oncogenomic abnormalities may also impact the response. 

More recently, the efficacy of tipifarnib was examined in a series of cell- and patient-derived xenograft models of head and neck squamous cell carcinoma (HNSCC) [40]. Genomic analyses have revealed that *HRAS* mutations occur in 6% of HNSCC at initial diagnosis [41] and in 15% of patients during acquisition of resistance to cetuximab [42], and *HRAS* mutations have been demonstrated to correlate with reduced response of HNSCC patients to cetuximab treatment [43]. Gilardi et al. reported that both tipifarnib and *HRAS* knockdown significantly reduced the growth of *HRAS* mutated cell lines with no effects observed in *HRAS* wild-type cells. The investigators also demonstrated that tipifarnib induced selective anti-tumor activity, with *HRAS* wild-type tumors growing progressively on tipifarnib treatment but *HRAS* mutant tumors being highly sensitive to tipifarnib when compared to control groups. In addition, tipifarnib significantly reduced angiogenesis, as shown previously [44,45,46], and inhibited cell cycle progression while inducing squamous cell differentiation. Indeed, the anti-tumor activity of tipifarnib shown by Gilardi and colleagues in these HNSCC *HRAS* mutant models was equivalent to or exceeded that reported with a combination of MAPK and PI3K inhibitors in a *HRAS* mutant lung cancer model [47]. Collectively, these findings highlight mutant *HRAS* as a targetable oncogene that can be inhibited by tipifarnib, resulting in either consistent stasis or tumor regression in vivo in multiple preclinical models. 

Despite these promising results, the clinical efficacy of tipifarnib during its initial evaluation in the late 1990s and early 2000s was limited and response rates were insufficient to support registrational trials. However, since its reintroduction to the clinic in 2015, findings from several trials have supported mutant *HRAS* as a target for the treatment of a subset of patients with HNSCC. Most recently, Ho et al. reported data from a Phase 2 clinical trial (KO-TIP-001, NCT02383927) investigating the efficacy of tipifarnib in second line and beyond recurrent and/or metastatic (R/M) head and neck squamous cell carcinomas, among others [48]. Patients received a starting dose of tipifarnib of either 600 or 900 mg administered orally twice daily on days 1–7 and 15–21 of 28-day treatment cycles until progression of disease or unacceptable toxicity. At the time of data analysis, 21 HNSCC patients with HRAS mutations with a variant allele frequency (VAF) of at least 20% had been treated with tipifarnib, of whom 18 were efficacy evaluable. The objective response rate among these evaluable patients was 50%; those patients that did not have an objective response did obtain a best overall response of stable disease. Progression-free survival on tipifarnib was 5.9 months versus 3.6 months on the patients’ most recent prior therapy. Safety was evaluated in all 30 treated HNSCC patients, regardless of VAF. The most frequently observed treatment-emergent adverse events (TEAEs) of any grade observed in >10% of patients were hematological-related events (anemia, neutropenia, leukopenia, lymphopenia) and gastrointestinal disturbances (nausea). Three patients experienced TEAEs leading to tipifarnib discontinuation. All three events were not related to tipifarnib and possibly related to disease. Based on this encouraging clinical activity, an international, multi-center, open-label, single-arm, pivotal study of tipifarnib after failure of platinum-based therapy in recurrent or metastatic HNSCC with *HRAS* mutations, AIM-HN, is under way (NCT03719690). Furthermore, encouraging results in urothelial carcinoma and salivary gland tumors were also reported. Twenty-four percent of HRAS mutant metastatic urothelial carcinoma patients treated with tipifarnib experienced an objective response. In addition, of 13 patients with recurrent/metastatic salivary gland tumors (SGT) treated with tipifarnib, one experienced an objective response and an additional seven patients had stable disease as best response. 

The recent completion of The Cancer Genome Atlas (TCGA) [41] has enabled the identification of patient populations harboring *HRAS* mutations that may benefit from tipifarnib therapy. Gilardi et al. performed a detailed analysis of genomic information in the TCGA database focused on revealing *HRAS* expression levels and mutational status in an array of cancer types [40]. The study showed that relatively few cancers harbor *HRAS* mutations, particularly thyroid cancer, pheochromocytoma and paraganglioma, and HNSCC, with HNSCC expressing the highest levels of *HRAS* transcripts. In agreement with previous findings, *HRAS* mutations are characterized, in most cases, by coincident loss-of-function mutations in caspase-8 and by the absence of *TP53* mutations. Moreover, *HRAS* mutant HNSCC cases are of low overall mutational burden and respond poorly to standard-of-care immuno-oncology therapies [41]. In summary, preclinical murine studies, in-depth oncogenic analyses, and ongoing clinical investigation in patients with mutant *HRAS* tumors may support tipifarnib as a novel precision therapeutic approach for HNSCC and other cancers. 

## 2. HRAS Dependency: Role of Unmutated HRAS in Progression and Chemoresistance in HNSCC

Driver oncoproteins are commonly hyperactive forms of signaling molecules that regulate cellular proliferation and survival. This hyperactivity may be achieved by mutation leading to constitutive activation and/or by overexpression of the wild-type protein due to fusion with a highly expressed gene, genetic amplification or transcriptional dysregulation. Given that activating point mutations render the protein activity independent of upstream signaling, it is not surprising that mutations of a given tumor driver pack a greater oncogenic punch than either amplification or overexpression of the wild-type form. However, the protein need not be mutated to represent a valuable therapeutic target. Indeed, increasing preclinical and clinical data suggest that targeting wild-type oncoproteins has potential therapeutic value in the era of personalized medicine, particularly in the context of the combination regimens that are increasingly becoming the standard of care in cancer therapy [49].

Oncogene amplification and overexpression are common phenomena in solid tumors, particularly in SCCs. For example, *KRAS* is mutated in approximately 30% of cases of lung adenocarcinoma (ADC) in TCGA’s PanCancer Atlas but amplified at a rate of only around 5%; in lung SCC, the relative frequencies are reversed (1% vs. 4%). Similarly, *EGFR* is mutated in twice as many lung ADCs as it is amplified, compared to a threefold excess of amplification vs. mutation in LSCC, where frequencies of high polysomy and amplification may be up to 40% [50]. *EGFR* is very rarely mutated in HNSCC but is amplified 10–30% of cases [51] and overexpressed at high frequency [52]. Perhaps the most compelling evidence for the importance of unmutated oncogenic driver proteins in SCCs is the approval and widespread use of the chimeric anti-EGFR antibody cetuximab as a standard of care treatment for HNSCC [53,54]. EGFR and MET are examples of receptor tyrosine kinases (RTK) that can become hyperactive through receptor clustering when overexpressed in HNSCC, but non-RTK oncogenes can also drive HNSCC, including *PIK3CA*, which is mutated and amplified at a higher prevalence, around 35%, in HNSCC [55], and the oncogenic chloride channel ANO1/TMEM16, a core element of the 11q13 amplicon, is found in a quarter of HNSCCs and more than half of ESCCs [56,57].

Despite—or perhaps because of—the high prevalence of *EGFR* amplification and overexpression in HNSCC, several clinical studies failed to demonstrate enhanced sensitivity to cetuximab in amplified or overexpressing populations [49,58]. Therefore, although cetuximab is the first and the only FDA-approved targeted therapy in HNSCC to date, the prescribing information makes no reference to EGFR. The picture is less clear in other SCCs: preclinical studies showed superior activity of cetuximab in *EGFR*-amplified and overexpressing esophageal SCC (ESCC) xenografts [59], and *EGFR*-amplified and overexpressing lung SCC patients responded better to gefitinib [49] and cetuximab and chemotherapy [54,60,61] than their EGFR^low^ counterparts. The FLEX trial compared chemotherapy (cisplatin and vinorelbine) with and without cetuximab in a cohort of first-line NSCLC patients, 35% of whom were SCC [61]. Addition of cetuximab to the regimen resulted in a 38% reduction in risk of death and a 2.3-month net increase in median survival among those with *EGFR* overexpression with no difference in overall survival (OS) among those with low *EGFR* expression [61]; but in a similar trial with a different cocktail of chemotherapeutics (paclitaxel and carboplatin), outcomes were not associated with EGFR mutation, increase in *EGFR* gene copy number, or EGFR protein [62].

**Figure 1:**
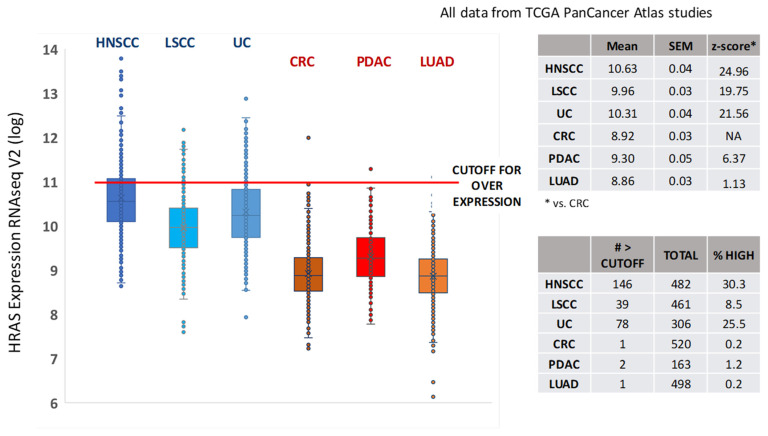
HRAS is overexpressed in squamous cell carcinomas. Data from TCGA PanCancer Atlas accessed at http://www.cbioportal.org/ (accessed on: 9 November 2020. Comparison of HRAS mRNA expression between the major adenocarcinomas, colorectal (CRC), pancreatic (PDAC), and lung (LUAD), and squamous cell carcinomas of the head and neck (HNSCC), lung (LSCC), and urothelium (UC). * vs. CRC.

To begin exploring the therapeutic potential of HRAS inhibition via farnesyltransferase inhibition beyond the HRAS mutant fraction of HNSCC, we reasoned that the biology of tumors driven by hyperactivity of wild-type oncoproteins is likely to resemble that of their corresponding mutant counterparts more than that of tumors with unrelated driver pathways. Intriguingly, several groups have reported that HRAS-mutant SCCs co-cluster in unbiased genomic and epigenomic profiling analyses. Indeed, genomic clustering suggests that HRAS mutations define a unique subset of HNSCC, characterized in most cases by coincident loss of function mutations in caspase 8 and enrichment for absence (near-mutual exclusivity) of TP53 mutations [40,55,63]. Furthermore, a recent systematic analysis of TCGA SCC cohorts by Campbell and colleagues reported that *HRAS*-mutant HNSCCs also cluster on the basis of copy number variations (CNVs, i.e. chromosomal alterations) and methylation pattern [64]. Closer inspection of TCGA PanCancer Atlas cohorts revealed that, although amplification at the *HRAS* locus is surprisingly rare, SCCs express significantly higher levels of HRAS than adenocarcinomas, and HRAS mRNA is overexpressed in around 30%, 25%, and 10% of cases of HNSCC, UC, and LSCC, respectively (Figure 1). The large majority of HRAS mutants are also found in the overexpressing population. Interestingly, the methylation cluster described in Campbell et al. is also significantly enriched for HRAS-overexpressing HNSCC cases, suggesting that HRAS expression levels could be used as a biomarker to explore the potential role of the wild-type form of the oncoprotein in HNSCC progression and drug resistance.

**Figure 2:**
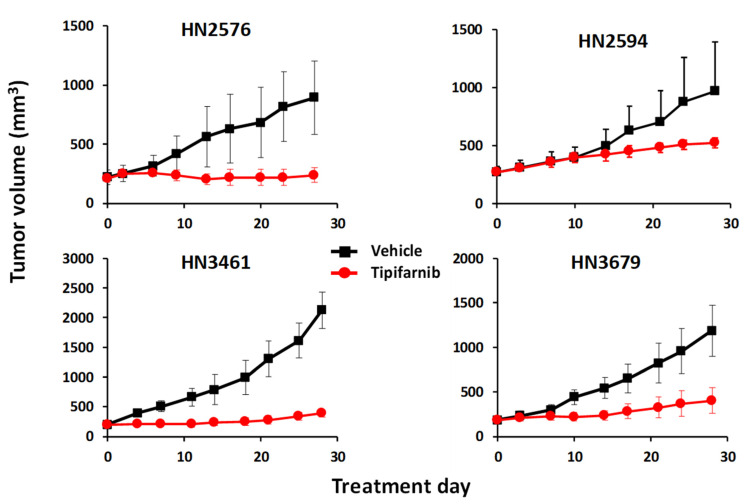
Some HRAS-overexpressing HNSCC PDX models are sensitive to tipifarnib. BALB/c nu/nu or SCID mice were inoculated subcutaneously with 2–3 mm tumor fragments, the PDX were allowed to establish to 250–350 mm^3^, the animals were randomized into groups of three and treated orally BID with vehicle or tipifarnib (60 mg/kg for SCID, 80 mg/kg for nu/nu) for 25–30 days. Tumor volumes were measured twice weekly in two dimensions using a caliper, and the volume was expressed in mm3 using the formula: V = (L × W × W)/2, where V is tumor volume, L is tumor length (the longest tumor dimension), and W is tumor width (the longest tumor dimension perpendicular to L).

We tested tipifarnib (80 mg/kg, BID) as a single agent in a panel of around 20 *HRAS* mutant and wild-type HNSCC patient-derived xenograft (PDX) models with mixed results. As reported previously [40], tipifarnib was highly active in the *HRAS* mutant setting and displayed weak activity in the majority of *HRAS* wild-type models, but we observed unexpectedly robust inhibition of tumor growth in a minority of *HRAS* wild-type cases, all of which expressed high levels of the *HRAS* gene (Figure 2). These hints of activity, while encouraging, were sporadic and variable in nature and did not extend to tumor regression, indicating that these tumors could tolerate HRAS depletion in isolation, but might also be rendered hypersensitive to other stressors. 

**Figure 3:**
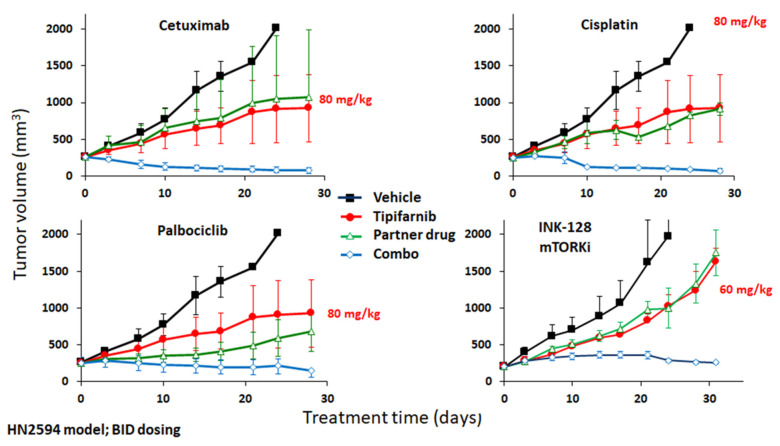
Tipifarnib displays additive or synergistic anti-tumor activity with a variety of partner drugs in HRAS-overexpressing patient-derived xenograft (PDX) models. BALB/c nu/nu or SCID mice were inoculated subcutaneously with 2–3 mm tumor fragments, the PDX were allowed to establish to 250–350 mm^3^ and the animals were randomized into groups of three before being dosed with vehicle or tipifarnib (60–80 mg/kg BID as labeled above) alone or in combination with cetuximab (1 mg/mouse IP QW), cisplatin (5 mg/kg Q3D PO), palbociclib (40 mg/kg QD PO), or INK-128 (2 mg/kg QD PO).

With the notable exception of Herceptin and other HER2 antagonists, few drugs directed against non-mutated oncoprotein targets have proven effective as single agents [43], but it is more likely that clinically-actionable dependencies on overexpressed wild-type drivers will emerge in the context of synthetic lethal interactions with other therapies. Indeed, cetuximab demonstrated enhanced activity in EGFR-amplified HNSCC PDX models in combination with fractionated irradiation [65]. Our preliminary data in HNSCC models spurred interest in a possible role of wild-type HRAS in innate resistance to standard-of-care drugs such as cisplatin and cetuximab as well as targeted agents in clinical development in HNSCC, including CDK4/6 inhibitors and PI3K pathway drugs. As shown in Figure 3, tipifarnib co-treatment sensitized the HRAS-overexpressing HN2594 PDX model to all four classes of drugs, inducing consistent regressions in all combination regimens, despite only the CDK4/6 inhibitor palbociclib being significantly active as a single agent in this experiment. In expanded tests, tipifarnib enhanced cisplatin activity in 3/12 HRAS wild-type models examined, all of which overexpressed the HRAS gene, as described previously [66]. Tipifarnib also increased tumor growth inhibition with palbociclib in the majority of HRAS^high^ models tested, but cetuximab was highly active in all four PDX studied, precluding assessment of this combination in these models; previous work suggests that HRAS signaling is a key driver of cetuximab resistance in experimental models and in the clinic [61]. In a third study focused on PI3K pathway combinations, synergy was noted with both the mTOR kinase inhibitor TAK-228 (sapanisertib) and the PI3Ka inhibitor BYL-719 (alpelisib), including in *HRAS* or *PIK3CA* mutant (20% of HNSCC) [67], *PIK3CA*-amplified (15%) [68] or HRAS-overexpressing (30%) (Figure 3) models, and this combination has previously been shown to be synergistic in CDX systems [69], suggesting that simultaneous blockade of these two prominent oncogenic pathways could offer potential benefit in a broad population of HNSCC patients.

In summary, extensive studies in panels of PDX models indicate that both mutant and overexpressed HRAS contribute significantly to the proliferation, survival, and innate drug resistance of HNSCC cells in vivo. HRAS is the predominant RAS isoform in squamous epithelial cells and the SCCs derived from them [55] and so may play a wider role. Although HRAS activity has previously been reported to contribute to most hallmarks of cancer [40] and to drive clinical resistance to cetuximab [42,43], it is also likely that HRAS-independent mechanisms contribute, at least in part, to the antitumor activity of tipifarnib in HNSCC models. Dozens of proteins are dependent upon farnesylation for membrane insertion and function [70]. In the next section, we explore the potential of farnesyltransferase inhibition to anchor combination regimens through mechanisms independent of HRAS.

## 3. Combination Approaches with FTIs in SCCs and Other Solid Tumors

Although RAS is known to play a key role in innate resistance to a variety of therapeutics used in SCCs, including platinum-based chemotherapy and anti-EGFR antibodies [43,66], and HRAS inactivation sensitizes HNSCC PDX tumors to a range of drugs in mice (Figure 3), it has been established that much of the documented antineoplastic activity of FTIs is mediated by effects on proteins other than RAS [71,72]. For example, several lines of evidence suggest that RHOB farnesylation may have contextual roles in tumor progression and survival. RHOB expression in Rat1 cells induces proliferation, which can be inhibited by FTIs [73]. RHOB has also been shown to be a direct regulator of phosphatase 2A (PP2A) activity via recruitment of the B55 subunit [74]. During lung cancer progression, downregulation of RHOB may inhibit PP2A activity, leading to activation of the Akt1-Trio-Rac1 axis, triggering cell migration and invasion. Furthermore, an AKT-dependent mechanism has been suggested to underlie RHOB-driven resistance to EGFR inhibitors in EGFR-mutant NSCLC models [75]. Another potential target of FTIs is RHEB (RAS homolog enriched in brain), a GTPase with two isoforms (RHEB1 and RHEB2) that are commonly upregulated in transformed cells and human cancer cell lines. RHEB binds and activates the mechanistic target of rapamycin (mTOR), a regulator of tumor cell growth, survival, and metabolism [76,77]. It is thought that mTOR is activated via farnesylation-dependent transient interactions of RHEB with the mTORC1 complex in lysosomal membranes [78]. Human RHEB1 and RHEB2 have been shown in vitro to be substrates for FTase, and treatment of cells with FTIs inhibits RHEB prenylation. Basso et al. demonstrated that treatment of MCF-7 cells with lonafarnib inhibited RHEB farnesylation, resulting in inhibition of DNA synthesis and S6 kinase activation. Furthermore, it was found that lonafarnib enhanced tamoxifen- and taxane-driven apoptosis, supporting the combination of FTIs with standard-of-care agents. RHEB-mTOR signaling has also been implicated in resistance to antineoplastic therapies [79]; thus, combination approaches may bypass these resistance mechanisms [80]. Recent findings by Mahkov et al. demonstrated that 786-O renal carcinoma cells expressing prenylation-incompetent RHEB display robust apoptosis in response to sunitinib treatment [81]. Moreover, the investigators examined the anti-tumor effect of sunitinib in combination with lonafarnib using mice bearing clear cell renal cell carcinoma (ccRCC) xenograft tumors. Monotherapy with either sunitinib or lonafarnib showed a moderate decrease in tumor growth; however, co-administration resulted in impressive reductions in tumor volume. Thus, FTIs may offer a means to circumvent sunitinib resistance, perhaps through prevention of RHEB localization to lysosomal membranes and subsequent downstream activation of mTOR signaling. 

Several additional families of proteins with functional roles in proliferation, invasion and other hallmarks of cancer are dependent upon farnesylation for appropriate intracellular localization and activity. Centromere protein-E (CENP-)E and CENP-F have also been shown to be FTI targets. CENP-E is a centromere-associated kinesin motor protein that functions in microtubule attachment to kinetochores, which is required for the separation of sister chromatids during mitosis [82,83]. CENP-F is a cell cycle-regulated passenger protein which also has mitotic function [84]. Both CENP-E and CENP-F are farnesylated proteins whose prenylation is inhibited by FTI treatment [85], subsequently preventing CENP-E association with microtubules and reducing levels of CENP-F at the kinetochores [85,86]. Similarly, the PRL family of protein tyrosine phosphatases (PTPS), also known as PTP-CAAX proteins, are a unique subfamily of PTPs that regulate cell growth and mitosis and have been shown to be upregulated in numerous human tumor cell lines and implicated in progression of several tumor types [87,88,89]. The PRL family includes three members, all of which are farnesylated. Al three forms traffic to the plasma membrane in transfected CHO cells, localization of which is inhibited by FTIs [90]. 

Some farnesylated substrates carry unknown significance for cancer but may still provide clinical utility, such as the DnaJ homologs which serve as co-chaperones and stimulate the ATPase activity of Hsp70, a cancer-associated protein [91]. One homolog, HDJ2, is a farnesylated protein whose prenylation status is used as a pharmacodynamic (PD) biomarker for FTase inhibition in clinical trials [92]. The functional significance of HDJ2 farnesylation remains unclear. Nuclear lamins (e.g., lamin A, lamin B), proteins that are required for nuclear envelope assembly, were some of the first proteins shown to be prenylated [93]. Similar to HDJ2, the functional consequence of lamin prenylation is unknown but may assist in the targeting of prelamins to the nuclear membrane. A mutation in *prelamin A* occurs in children with Hutchinson-Gilford progeria syndrome (HGPS), a debilitating and fatal disease characterized by premature aging. FTIs have been shown to reverse the abnormal nuclear phenotype in cells derived from HGPS patients [94], and the FTI lonafarnib was recently approved for therapy of this devastating rare disease [95]. 

In some instances, farnesyltransferase inhibition has been found to mediate antitumor activity, but the farnesylated substrate underlying the effect remains to be confirmed. For instance, tipifarnib and other FTIs have been suggested to act as antiangiogenic agents in several tumor types including HNSCC [40,46], but the mechanisms are yet to be elucidated [44] and may be hard to delineate from indirect downstream consequences of inhibiting another target, such as HRAS [40]. Similarly, FTIs have been shown to rapidly trigger the production of reactive oxygen species (ROS) [96]. Though the mechanism of ROS generation remains unclear, the consequences include DNA damage responses, such as activation of DNA repair proteins and induction of RHOB [96]. In turn, RHOB may sensitize cancer cells to DNA damage-induced apoptosis following genotoxic stress [97]. FTIs may also offer the potential to modulate antitumor immunity. RAS-MAPK signaling drives expression of the *CD274* gene leading to PDL-1 overexpression [98] but also downregulates MHC Class I expression, reducing immunogenicity and undermining the effectiveness of immune checkpoint inhibitors [99]. Therefore, FTI treatment might enhance responsiveness of HRAS-dependent SCCs to immunotherapy. 

CXCL12 (or SDF1) is a potent immunoregulatory chemokine and CXCL12-CXCR4 signaling is associated with resistance to immunotherapy in HNSCC [100]. Intriguingly, we have recently reported that CXCL12 production by stromal fibroblasts, the predominant CXCL12-producing cell in solid tumors, can be inhibited by tipifarnib in vitro [101]. Further studies are ongoing in our laboratory to characterize this novel FTI activity and the potential of FTIs to enhance immunotherapy in SCC models. CXCL12 also shows promise as a biomarker guiding FTI therapy in T-cell lymphoma patients. Recently, the effect of tipifarnib on the CXCL12 axis was investigated in an open-label, Phase 2 study in relapsed or refractory peripheral T-cell lymphoma [102]. Tumor gene expression data were available for 12 of the 18 evaluable patients. Five of those patients had elevated CXCL12 expression and experienced tumor size reductions and >6-month median time to progress following tipifarnib treatment. Thus, tipifarnib may be a promising therapeutic approach in this patient population.

It is likely that protein farnesylation plays an actionable role in many oncogenic signaling pathways. Indeed, there are hundreds of proteins with CAAX motifs that are potentially farnesylated [72], although the true number of farnesylation-dependent proteins is probably several dozen in most cell types [70]. Given this pleiotropy, it is perhaps counterintuitive that tipifarnib has been evaluated in more than 5000 patients and has been generally well-tolerated with a clearly delineated toxicity profile when used at doses that sharply reduce farnesylation of several PD biomarkers in vivo [92]. Several mitigating factors, including redundancy with farnesylation-independent orthologs or collateral signaling pathways, varying sensitivity to FTase activity between substrates [70], and pharmacokinetic compartmentalization, may render FTIs more selective in vivo, but mounting evidence supports the notion that farnesylated target oncoproteins including HRAS, RHEB, and RHOB could be exploited as part of FTI-anchored combination regimens in SCCs and a range of other tumor types.

## 4. Conclusions

In summary, preclinical murine studies, in-depth oncogenic analyses, and ongoing clinical investigation in patients with mutant HRAS tumors may support tipifarnib as a novel precision therapeutic approach for HNSCC and other cancers. Recent advances in genomic and cellular studies have led to the identification of HRAS mutations as drivers of tumor growth in a subset of HNSCC. These mutations in an oncogene that is uniquely sensitive to inhibition of farnesylation appear to sensitize the tumors to farnesyl transferase inhibitors such as tipifarnib, as supported by animal models and ongoing clinical trials. In addition, preclinical studies in PDX models overexpressing HRAS have demonstrated that tipifarnib sensitizes these tumors to several drugs in clinical use in SCCs, suggesting that the benefit of tipifarnib may be extended to include those patients with HRAS overexpressing tumors when used in combination with drugs such as cetuximab, alpelisib, and cisplatin. Furthermore, inhibition of other farnesylated proteins, such as RHEB and RHOB, may help overcome resistance to standard therapies in SCCs and additional tumor types.
